# LPA-MNI: An Improved Label Propagation Algorithm Based on Modularity and Node Importance for Community Detection

**DOI:** 10.3390/e23050497

**Published:** 2021-04-21

**Authors:** Huan Li, Ruisheng Zhang, Zhili Zhao, Xin Liu

**Affiliations:** School of Information Science and Engineering, Lanzhou University, Lanzhou 730000, China; lih17@lzu.edu.cn (H.L.); zhaozhl@lzu.edu.cn (Z.Z.); xinl@lzu.edu.cn (X.L.)

**Keywords:** community detection, randomness, label propagation, modularity, node importance

## Abstract

Community detection is of great significance in understanding the structure of the network. Label propagation algorithm (LPA) is a classical and effective method, but it has the problems of randomness and instability. An improved label propagation algorithm named LPA-MNI is proposed in this study by combining the modularity function and node importance with the original LPA. LPA-MNI first identify the initial communities according to the value of modularity. Subsequently, the label propagation is used to cluster the remaining nodes that have not been assigned to initial communities. Meanwhile, node importance is used to improve the node order of label updating and the mechanism of label selecting when multiple labels are contained by the maximum number of nodes. Extensive experiments are performed on twelve real-world networks and eight groups of synthetic networks, and the results show that LPA-MNI has better accuracy, higher modularity, and more reasonable community numbers when compared with other six algorithms. In addition, LPA-MNI is shown to be more robust than the traditional LPA algorithm.

## 1. Introduction

All kinds of complex systems can be described as networks, such as biology network, social network, collaboration network, and World Wide Web, etc. Community structure often hides in the network. Community structure refers to a group of nodes that are similar to each other, but different from that of other parts in the network. In other words, the nodes within a community structure are tightly connected while the connections are relatively sparse among community structures [1]. Finding and analyzing community structures is of great significance for understanding the complex network. Community detection has been used in the recommendation system [2] and influence maximization problem [3].

In recent years, the study of community detection has attracted a lot of attention and many algorithms have been proposed. The pioneering work of community detection was the GN algorithm that was proposed by Girvan and Newman [4], which was based on the idea of partition. The basic principle of partition-based method is to find out all of the links between communities and delete them, and then each connected branch corresponds to a community. And many related works inspired by GN have been proposed [5,6,7,8]. In particular, Newman and Girvan [1] proposed a quantitative standard called modularity function for depicting the quality of the community structure. This function gives a clear definition of community structure and it has achieved great success in practical applications. Therefore, it has been gradually accepted. Meanwhile, methods that adopt modularity function as optimization function have become mainstream for community detection. For example, Fastgreedy [9], BGLL [10], simulated annealing method [11], and spectral analysis algorithm [12,13]. However, these methods that are based on modularity function may fail to identify the community structure whose size is smaller than a certain size, which is called the resolution limit problem [14]. In order to deal with the problem, some modifications of the modularity function have been proposed [15,16,17,18]. In addition, heuristic algorithms [19,20] that were based on Markov random walk theory [21] were also proposed to identify communities. Additionally, a community detection algorithm, combined with random walk and MapReduce parallel programming framework, was proposed in [22]. Furthermore, meta-heuristic algorithm has also gradually become a competitive method, which has attracted many researchers’ attention. Community detection methods that are based on ant colony algorithm [23], genetic algorithm [24], particle swarm optimization algorithm [25], bat algorithm [26], and whale optimization algorithm [27] have been proposed. Although these algorithms can obtain good partition results, their efficiency cannot be guaranteed with the increase of the dataset. In recent years, with the development of machine learning technology, network representation learning [28] has become a new research field. The community detection algorithm, combined with network representation learning, has also been developed [29]. Additionally, the algorithm was based on deep learning was proposed [30]. As a valid method in unsupervised learning, Nonnegative Matrix Factorization (NMF) has also been gradually applied to analyze community structure [31,32]. Although the algorithm that is based on NMF has good interpretability, it usually needs the prior knowledge of the number of communities in the network, but the number of communities is generally unknown. Community detection algorithms that are based on network topology [33,34,35,36,37] have also been proposed recently. Other community detection algorithms were mentioned in [38,39].

Different from the modularity optimization algorithm, some of the algorithms based on heuristic strategy can detect community without a specific objective function and be designed through an intuitionistic and enlightening idea. The label propogation algorithm proposed by Raghavan et al. [40] adopts simple label propagation [41] to identify community structures. The heuristic rule of the label propagation method is that any nodes in a community structured network should be in the same community with most of their neighbors. This heuristic algorithm only uses network structure as input without setting free parameters. The label propagation algorithm assigns the unique label to each node and keeps updating the label of each nodes according to its neighnors until no label is changed. The biggest advantage of LPA is the linear computational complexity, but its uncertainty and randomness are prominent. Different community structures may be generated in different runs because of its random strategies in initialization and updating orders. Recently, many extended LPA have been proposed to optimize the performance [42,43,44,45,46]. However, these extensions cannot completely solve the randomness problem, or they greatly improve the complexity of the algorithm.

In this paper, by combining the modularity function and node importance with the original LPA, an improved algorithm, termed as LPA-MNI, is proposed to overcome the randomness of LPA. Firstly, LPA-MNI employs modularity optimization procedures to identify initial communities. Secondly, all of the nodes in one initial community are assigned the same label. Subsequently, in the iterative processes of label propagation, LPA-MNI determines the node order of label updating according to the descending order of node importance. When more than one label is contained by the maximum number of nodes, LPA-MNI calculates the importance of each node and then selects the most influential node’s label to update. Finally, extensive experiments with comparative algorithms on real-world and synthetic networks have shown that LPA-MNI has an improvement of performance on community detection. Additionally, LPA-MNI can effectively overcome the randomness and inaccuracy of the LPA algorithm.

## 2. Related Work

The graphs that are discussed in this paper are simple, undirected, and unweighted networks. Let G(V,E) be a graph, where V(G) denotes the node set, E(G) denotes the edge set. The number of nodes is |V(G)|=n and the number of edges is |E(G)|=m.

**Figure 1:**
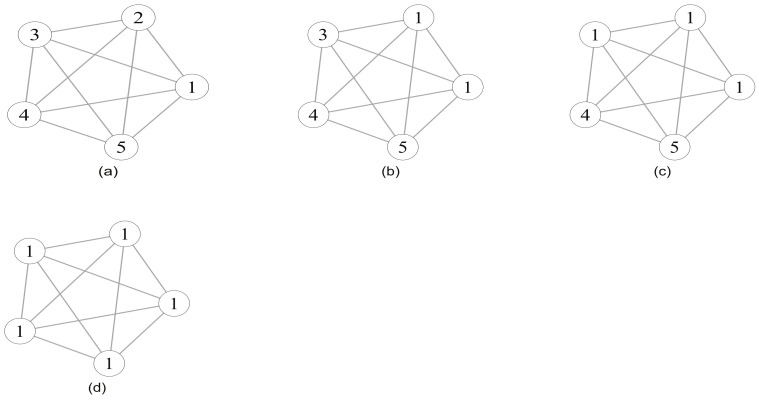
Illustration of label propagation. Labels are updated one by one from (**a**–**d**). Due to the high density of edges, all nodes acquire the same label.

Raghavan et al. [40] first applied the idea of label propagation in graph classification to community detection and proposed the famous label propagation algorithm. The algorithm does not require priori knowledge, the number of communities that the network should be partitioned into, and need not define functions determining when to stop iteration. At the beginning, suppose that every node in network has a label indicating their attributive community, and then each node updates its label according to the label with maximum number in its neighbors. As the labels propagate, the tightly connected individual in the network can quickly reach a stable state with a unique label (Figure 1), and the nodes with the same label are considered to belong to the same community structure. Algorithm 1 provides the process of label propagation algorithm.

LPA requires neither prior knowledge of the number of community nor functions as a condition for algorithm convergence. Moreover, the time complexity of the algorithm is near-linear. Therefore, LPA has become one of the most classical algorithms, and it has been widely accepted and used.
**Algorithm 1:** LPA.**Input:**G=(V,E)**Output:** The result of community detection1:Initializaion: assign a unique label to each node in the network, $C_{x}\left(0\right)=x$2:Set *t*=13:Arrange nodes in random order and set it to *X*.4:Select each node *x* in the *X* sequentially and update its label according to the following function, $C_{x}\left(t\right)=f\left(C_{x_{i1}}\left(t\right),\ldots ,C_{x_{im}}\left(t\right),C_{x_{i(m+1)}}\left(t-1\right),\ldots ,C_{x_{ik}}\left(t-1\right)\right)$, where $x_{i1},\ldots ,x_{im}$ are the neighbors that have been updated before time *t*, and $x_{i(m+1)},\ldots ,x_{ik}$ are the neighbors that have not been updated before time *t*.5:If the label in network no longer changes, stop the algorithm, else set t=t+1 and go to step 3.

Barber et al. [47] considered LPA as an optimization problem, and give the corresponding objective function. By studying the characteristics of the objective function, they revealed the defects of LPA in principle and practical applications. Most importantly, during the operation of the algorithm, the increase of the objective function does not necessarily mean the improvement of quality of the community. In order to overcome this shortcoming, they modified the objective function and designed a constrained label propagation algorithm, named LPAm (modularity-specialized label propagation algorithm). It is interesting that the modified objective function is exactly the modularity function *Q*, and the improved algorithm LPAm corresponds to modularity function optimization.

Liu et al. [48] found that LPAm has the characteristic that the number of nodes in each community is similar, which is to say, the algorithm has a tendency to fall into local optimum. To jump out the local optimum, they gave a multistep greedy agglomerative algorithm (MSG). Subsequently, they combined the algorithm LPAm with the MSG, and proposed a modularity optimization and hierarchical label propagation algorithm LPAm+, which leaves the clustering performance of the label propagation algorithm further improved.

Xie et al. [49] found that, after five iterations, ninety-five percent of the nodes can be correctly clustered by LPA. According to this discovery, they improved the update and iteration rules in the LPA algorithm, which greatly reduces the unnecessary update and iteration processes in the original algorithm, especially when dealing with complex network structures, and the efficiency of the algorithm is greatly improved.

Cordasco et al. [50] proposed a semi synchronous LPA algorithm, which makes any two adjacent nodes not have the same color by parallel coloring to the network nodes, and propagates the labels simultaneously.

Gui et al. [51] proposed an improved LPA algorithm that was based on community belonging degree, named LPA-CBD, which can overcome the randomness of the original algorithm and determine the center node of each community. However, the time complexity of the algorithm is $O(n^{2})$, and it is tremendously increased when compared with the near-linear time complexity of the original algorithm.

Xing et al. [44] proposed a novel label propogation algorithm for community detection, called NIBLPA. The algorithm considers both the *k*-shell value and the degree of node itself as well as its neighbors’ *k*-shell values to calculate the node improtance of every node. Subsequently, NIBLPA fixes the node updating order in the descending order of node importance value. However, the algorithm introduces a parameter alpha, which brings uncertainty to the community detection results.

Zhang et al. [45] replaced the method of calculating the node importance of NIBLPA algorithm with the Bayesian network. Although it can get stable results by avoiding the randomness in label propogation process, the algorithm also needs to adjust the parameter alpha. Other improved LPA algorithms, such as COPRA [52] and SLPA [53], have also been put forward for community detection in complex network.

However, these improved methods cannot completely solve the randomness problem, or they improve the complexity of LPA. Overcoming the instability and maintaining the efficiency of the original LPA algorithm still need to be explored. Therefore, in this study, modularity and node importance are applied to the improvement of LPA.

## 3. Methods

**Figure 2:**
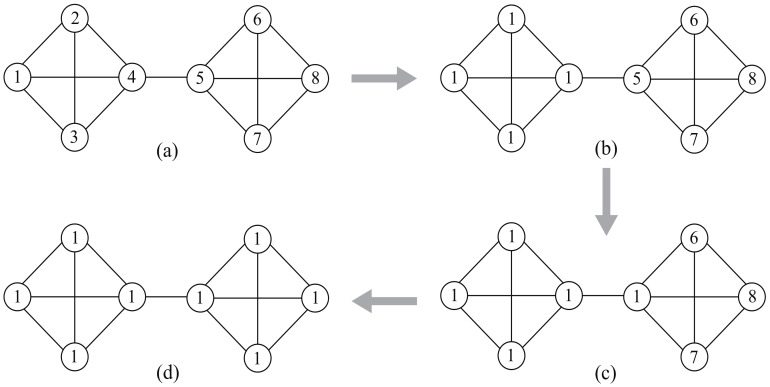
(**a**) A sample network; (**b**,**c**) a partion result at some step; and, (**d**) a failed partion result.

The sample network shown in Figure 2 illustrates that randomness will affect the accuracy of LPA. Initially, each node has a unique label (Figure 2a). Suppose that, at some step, the nodes in the left community have shared the same label 1, while the nodes in the right community still have unique labels 5–8 (Figure 2b). If node 5 randomly selects label 1 as its new label (Figure 2c), then all of the nodes may eventually be divided into the same community (Figure 2d). The random strategies implemented in the algorithm lead to the randomness of LPA.

**Figure 3:**
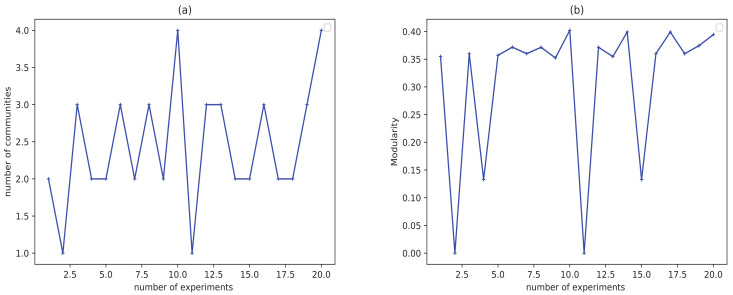
The results of LPA on karate network for 20 times. (**a**) The number of communities obtained in 20 experiments. (**b**) The values of modularity obtained in 20 experiments.

Figure 3 displays the result of community detection by LPA on Zachary karate club network [54]. It can be concluded that the number of communities and modularity in twenty experiments are fluctuant, which proves the division result of LPA is unstable and inaccurate.

In this paper, we propose an improved label propagation algorithm based on modularity and node importance (LPA-MNI) in order to solve the instability problem of LPA. The algorithm first discovers rough community of the network, and then assigns same labels to all nodes in the same rough community. Finally, implement the label propagation for community detection.

### 3.1. Rough Community Detection

Modularity that is defined by Newman and Girvan [1] is the most often used function to measure the result of community partition. It is defined, as follows:

(1) $$Q=\frac{1}{2m}\sum_{i,j}\left[A_{i,j}-\frac{k_{i}k_{j}}{2m}\right]\delta \left(C_{i},C_{j}\right)$$

where $A_{i,j}$ is the adjacency matrix of network, $k_{i}$ represents the degree of node *i*, *m* is the number of edges in a network, $C_{i}$ is the community to which node *i* is assigned, the function δ(u,v)=1 if u=v and 0 otherwise. A greater value of Q means a denser connection in the partition.

The first stage of our algorithm is to discover rough community structure by modularity. Assume that we start with a simple network of *N* nodes. Firstly, we assign different communities to each node. After this initialization, the number of communities in the network is equal to the number of nodes. Subsequently, for each node *i*, we remove *i* from its own community and place it in the community of its neighbor *j*, evaluating the modularity gain at the same time. The node *i* will join the community of *j*, for which this gain is positive and maximum. If there is no gain to be satisfied, node *i* stays in its original community. Such a merging process is applied repeatedly and sequentially for all nodes until no further improvement can be achieved. The first phase of the algorithm will reach the local maximum of modularity function. It should be noted that the order of nodes has great influence on the results, especially the computation time. Therefore, the degree centrality [55] is used to arrange the nodes in our algorithm in order to avoid some nodes with sparse links being considered several times during the merging process. Whether each node *i* merges with its neighbor *j* depends on the value of modularity function that is related to the node degree and the number of edges in the network, and these two values are fixed. Accordingly, for fixed iteration order, the initial community structure that is obtained by each iteration is constant. Equation (2) calculates the modularity before *i* is moved out from its own community, and Equation (3) calculates the modularity after moving *i* into a neighbor community. $\sum_{in}$ in equation represents the sum of the edges in community *C*, $\sum_{tot}$ is the sum of the edges incident to nodes in *C*. It is obvious that the gain in modularity ΔQ can easily be computed by $Q_{1}-Q_{2}$.

(2) $$Q_{1}=\frac{\sum_{in}}{2m}-{\left(\frac{\sum_{tot}}{2m}\right)}^{2}+\frac{0}{2m}-{\left(\frac{k_{i}}{2m}\right)}^{2}$$

(3) $$Q_{2}=\frac{\sum_{in}+k_{i,in}}{2m}-{\left(\frac{\sum_{tot}+k_{i}}{2m}\right)}^{2}$$

In this process, the nodes in the network will be divided into rough communities based on the modularity function. Subsequently, we assign the same label to nodes in the same community rather than assigning unique label to each node by LPA. This improvement can significantly reduce the number of labels in the network and further avoid the randomness in selecting labels of nodes according to their neighbors in subsequent iterations, which is helpful for dealing with the instability of LPA. In addition, the proposed strategy is in favor of reducing the number of iterations in label updating.

### 3.2. Label Update Strategy

When compared with other nodes, the important nodes in a network can affect the structure of the network to a greater extent. The number of important nodes is small, but their influences can be quickly transmitted to most nodes in the network [56]. The importance of node is also called centrality, which refers to that the importance of node is equivalent to the connection among nodes [57]. There are many strategies for measuring the importance of node, and the degree centrality [55] is widely applied because of its simplicity, intuition, and low computational complexity. The degree centrality depicts the direct influence of node. It is believed that the greater degree a node has, the more it directly affect its neighbors, and the more important it is. The normalized degree centrality index of node *i* is defined, as follows:

(4) $$DC\left(i\right)=\frac{k_{i}}{n-1}$$

where $k_{i}=\sum_{i}a_{ij}$, $a_{ij}$ is the element of the *i* row *j* column in the network adjacency matrix *A*, *n* is the number of nodes in the network, and (n−1) is the maximum possible degree value of the node.

As mentioned before, LPA applies three random strategies in updating labels, which leads to the randomness of the result. LPA-MNI uses the node importance assessment method (Equation (4)) to avoid the instability. During the iteration, LPA-MNI updates the nodes in descending order according to the importance of each node. When the number of more than one label reaches the maximum, the proposed alaorithm calculates the importance of each label and selects the label with the largest importance to update the node label. The above two steps effectively solve the randomness problem in LPA and, thus, the result of LPA-MNI is deterministic and accurate.

### 3.3. The Framework of LPA-MNI Algorithm

Algorithm 2 provides the details of LPA-MNI.
**Algorithm 2:** LPA-MNI.**Input:**G=(V,E)**Output:** The result of community detection1:Initialize a community to each node of the network.2:Cancluate the node importance of all nodes according to Equation (4).3:D← Arrange the nodes according the node importance.4:For each node i∈D, remove *i* from its own community and place it in the community of neighbor *j*, and compute the gain of modularity during the process. Place the node *i* in the community for which this gain is maximum. The process is executed repeatedly and sequentially for all nodes in order *D* until the gain is unchanged and the initial rough communities are obtained.5:Assign same label to the node in the same initial community defined by step 4.6:Set *t* = 1.7:For each i∈D, update its label according to the following function, $C_{x}\left(t\right)=f\left(C_{x_{i1}}\left(t\right),\ldots ,C_{x_{im}}\left(t\right),C_{x_{i(m+1)}}\left(t-1\right),\ldots ,C_{x_{ik}}\left(t-1\right)\right)$, when the number of more than one label reaches maximum, the importance of neighbor nodes is calculated (Equation (4)), and the most important node’s label are assigned to the current node.8:If the labels in the network become steady, stop the algorithm. Else, set t=t+1 and go to step 7.

### 3.4. Computational Complexity

The computational complexity of the proposed algorithm is discussed here. The algorithm consists of several independent phases. In the first stage, the time complexity of initializing each node as an independent community is represented as O(n). For the process of discovering rough communities, the complexity is denoted by O(n∗k), in which *k* represents the average degree of the network. The time complexity of computing importance of all nodes is O(m), and that of the process of ranking nodes according to degree centrality can be expressed as O(nlogn). In the worst case, the time complexity of updating the labels for the remaining nodes is O(n∗k). Consequently, the time complexity of the proposed algorithm is O(n)+2∗O(n∗k)+O(m)+O(nlogn)≈O(m+nlogn).

## 4. Results and Discussion

In order to evaluate the performance of our proposed algorithm LPA-MNI, several experiments are conducted on real-world and synthetic networks. The performance of LPA-MNI is compared with other state-of-the-art methods, i.e., Fastgreedy [9], LPA [40], Leading eigenvector [6], Walktrap [19], an improved LPA algorithm NIBLPA [44], and EdMot [58].

### 4.1. Evaluation Metrics

In addition to the modularity that is discussed in Section 3.1, Normalized Mutual Information (NMI) [59] is also employed to evaluate algorithm performance. In fact, these evaluation metrics are widely used to measure the performance of clustering algorithm. For two partitions *A* and *B* of a network, the value of NMI is computed by the following equation:

(5) $$NMI\left(A,B\right)=\frac{-2\sum_{i=1}^{C_{A}}\sum_{j=1}^{C_{B}}N_{ij}\log\frac{N_{ij}N}{N_{i}N_{J}}}{\sum_{i=1}^{C_{A}}N_{i}\log\frac{N_{i}}{N}+\sum_{j=1}^{C_{B}}N_{J}\log\frac{N_{j}}{N}}$$

where $C_{A}$ and $C_{B}$ denote the number of communities of partition *A* and *B*, *N* represents the total number of nodes in the network, and $N_{ij}$ represents the number of the same nodes in the community *i* of partition *A* and the *j*th community in partition *B*. $N_{i}$ is the sum of the row *i* of matrix $N_{ij}$ and $N_{j}$ refers to the sum of the column *j*. In this experiments, partition *A* represents the real community of the network and partition *B* represents the community discovered by algorithms. The value of NMI(A,B) ranges from 0 to 1, where NMI(A,B)=0 when partition *A* and *B* are completely different. If partition *A* exactly corresponds to partition *B*, then NMI(A,B)=1.

Adjusted Mutual Information (AMI) [60,61] is an adjustment of the Mutual Information (MI) score to account for chance. AMI augments NMI’s consistent upper bound (1.0) with a consistent zero expectation to adjust for chance clusterings. When compared with NMI, AMI can also calculate the similarity between two clusters, but its value range is between −1 and 1. The NMI metric has been criticized as not fitting for weak communities. We also use AMI to measure the performance of the partition results.

### 4.2. Experiments on Real-World Networks

Firstly, the experiments are conducted on some real-world networks in which the ground truth communities’ membership is already known. Subsequently, other experiments are carried out on other real-world networks with unknown community structure. The paremeter alpha of NIBLPA is set to 0.5. LPA is processed 100 times and the average value is used as the results in all of the experiments because of its randomness. We also analyze the fluctuation range of all results in order to compare the stability of algorithms. Table 1 shows the topology features of real-world networks that were used in this paper. Ca_Hep, Astro-ph, Cond_mat, and Cond_mat2005 are downloaded from arXiv (www.arxiv.org/) (accessed on 18 April 2021). The other networks are downloaded from website (http://www-personal.umich.edu/~mejn/netdata/) (accessed on 18 April 2021).

#### 4.2.1. The Networks with Known Community Structure

Zachary Karate Club network is the most commonly used network for community detection, and it is composed of 34 nodes and 78 edges. Each node and each edge represent a member of the club and the interaction between members, respectively. The conflict between president (node 34) and instructor (node 1) causes 34 members of the club to be divided into two clusters. Table 2 shows the actual community structure of karate. Table 3 illustrates the experimental results of seven algorithms on karate network and, for each instance, the best modularity, NMI and AMI are presented in boldface. The modularity, NMI, and AMI of LPA are in the form of $avg_{value}\pm \left(max_{value}-avg_{value}\right)$. ($avg_{value}$ and $max_{value}$ represent the average value and maximum value of 100 times.)

**Figure 4:**
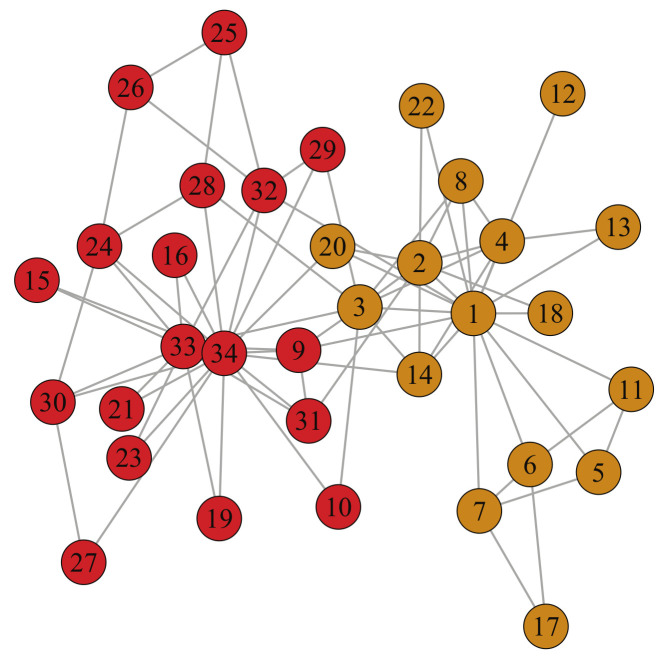
The result of the LPA-MNI algorithm on karate network.

LPA-MNI successfully detects two communities and accurately matches the actual community structure, as shown in Figure 4. However, the number of communities (CN) obtained by Fastgreedy, Leading eigenvector, Walktrap, NIBLPA and EdMot are 3, 4, 5, 3, and 3, respectively (Table 3). The NMI and AMI value of LPA-MNI algorithm are 1, which are the best value when compared with other algorithms especially LPA with uncertainty. In addition, our algorithm has better modularity (Q) than Walktrap and NIBLPA.

**Figure 5:**
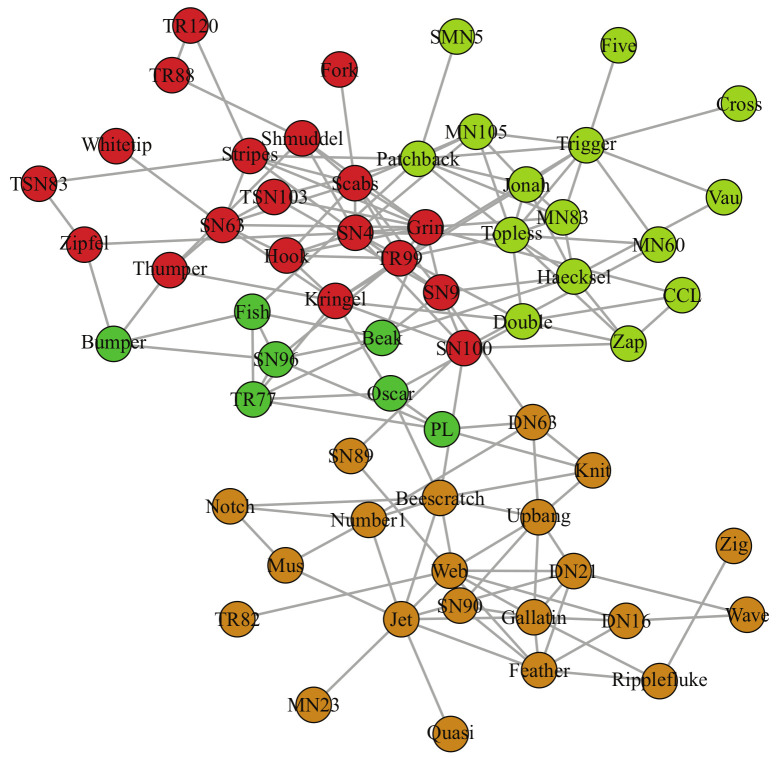
The result of LPA-MNI algorithm on dolphins network.

The Bottlenose Dolphins network consists of 62 nodes and 159 edges reflecting the social behavior of dolphins. It was initially thought to be divided into two communities [62], while further research [69] shows that the network should be divided into 4 communities, which can clearly reflect the social relations between dolphins. Figure 5 shows the community structure detected by LPA-MNI and four communities are accurately segmented.

Table 4 shows the results of seven algorithms on dolphins network and, for each instance, the best modularity, NMI, and AMI are presented in boldface. The modularity (Q = 0.527) and accuracy (NMI = 0.843, AMI = 0.833) of LPA-MNI are better than those of other six algorithms, as shown in Table 4.

The American College Football network represents the network of the 2000 regular season football match in the United States. It consists of 115 nodes that represent teams and 613 edges representing the matches between teams. The 115 teams ought to be divided into 12 communities [4], and each team has more competitions in its own community than those in other communities.

**Figure 6:**
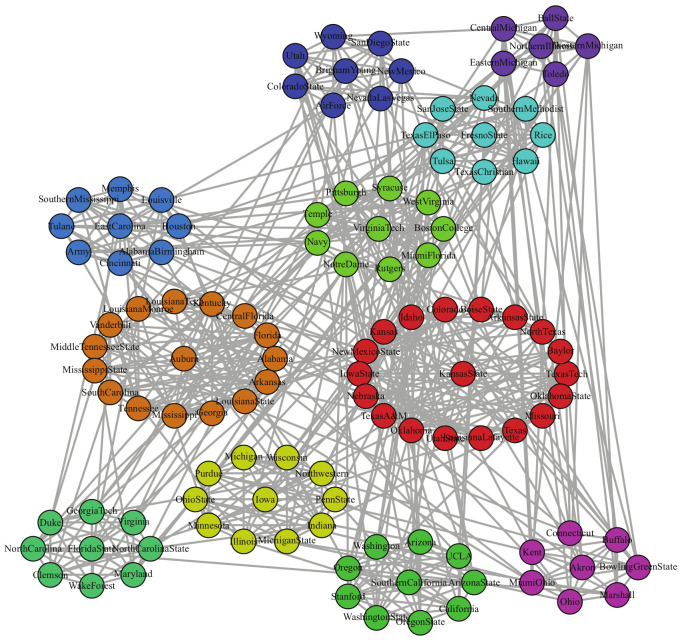
The result of LPA-MNI algorithm on football network.

11 communities, as shown in Figure 6, are obtained by LPA-MNI algorithm, which outperforms Fastgreedy (six communities), Leading eigenvector (eight communities), Walktrap (ten communities), NIBLPA (nine communities), and EdMot (nine communities). Table 5 shows the experiment results of seven algorithms on football and the better Q, NMI, and AMI are in boldface. The NMI of LPA-MNI and EdMot are greater than that of other five methods. In addition, modularity shows that LPA-MNI is superior to other comparison algorithms besides Walktrap and EdMot.

The LPA-MNI algorithm obtains good community partition results in three networks with known community structure. Especially in the karate network, the network is divided into two communities because of the conflict between president and instructor. The LPA-MNI algorithm can find two communities accurately. These three networks all reflect the real-world problems. The above results show that the LPA-MNI algorithm can deal with the community partition in real problems.

**Figure 7:**
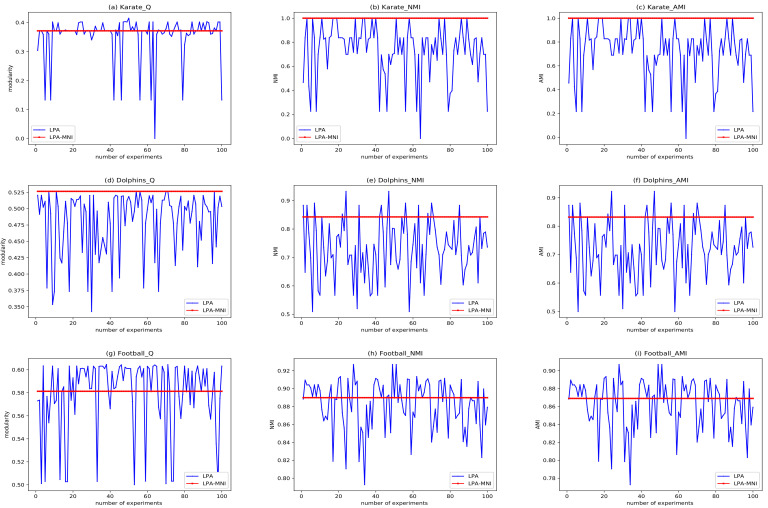
Comparisons of LPA and LPA-MNI.

In order to show the improvement of the proposed algorithm in stability, the experimental results of LPA-MNI and LPA for 100 times on karate, dolphins, and football are displayed in Figure 7. It can be seen from Figure 7a–c that the modularity (Q), NMI, and AMI of LPA on the karate network in 100 experiments are fluctuant, while Q, NMI, and AMI of LPA-MNI in each experiment are 0.372, 1, and 1 respectively, which is very stable. Figure 7d–f display that the modularity of LPA fluctuates between 0.35–0.50, NMI fluctuates between 0.5–0.9, and AMI fluctuates between 0.5–0.9. The results reveal that LPA-MNI is a more robust method when compared with LPA on the dolphins network. Figure 7g–i exhibits that, on the football network, LPA-MNI has a stable value in each experiment, yet LPA is unstable. Information entropy [70] is used to reflect the uncertainty of information sources. Therefore, we calculate the information entropy of Q, NMI, and AMI sequences that were obtained by the two algorithms. The information entropy of Q, NMI, and AMI obtained by LPA on karate network are as follows: 3.941, 3.830, and 3.830. Additionally, on the dolphins network, they are: 5.612, 5.660, and 5.660. The results obtained on the football network are as follows: 5.597, 5.937, and 5.937. For LPA-MNI, the information entropy of its results on all networks are 0. Therefore, it can be concluded that LPA-MNI significantly improves the stability of community detection.

#### 4.2.2. The Networks with Unknown Community Structure

LPA-MNI is also tested on real-world networks with unknown community structure. For these datasets, we only investigate the modularity, because the number of communities is unknown. In addition, LPA is processed 100 times and the average value is used as the results because of its randomness.

Table 6 shows the experimental results of LPA-MNI and other algorithms on these datasets for comparision, and the better modularity are presented in boldface. The modularity of LPA are in the form of $avg_{value}\pm \left(max_{value}-avg_{value}\right)$ ($avg_{value}$ and $max_{value}$ represent the average value and maximum value of 100 times.) It is obvious that LPA-MNI has the highest value of modularity in the datasets Riskmap, PolBlogs and Astro-ph. Regarding remaining datasets, LPA-MNI obtains better modularity than LPA, Leading eigenvector, Walktrap, and NIBLPA, but it is similar to the Fastgreedy algorithm. However, LPA-MNI is based on a heuristic strategy and the goal is to find reasonable community structure. As is known to all, the Fastgreedy algorithm is based on the idea of modularity and it may suffer from resolution limit problems that make it impossible to identify small communities. The Fastgreedy algorithm merges small communities to obtain higher modularity value, so the number of communities detected by Fastgreedy algorithm is smaller than Walktrap and LPA-MNI. As for NIBLPA, although the performance is relatively stable, its results are worse than LPA-MNI. In addition, NIBLPA needs to adjust the parameter alpha. LPA-MNI can steadily detect communities, while the results of LPA are fluctuant. When compared with EdMot algorithm, LPA-MNI obtains better community partition results, except for network Riskmap, Jazz, and Yeast. The experimental results on networks with an unknown community structure show that the LPA-MNI algorithm can achieve relatively stable and accurate community partition results. Most of all, LPA-MNI can return more stable and satisfing results when compared with the original LPA.

### 4.3. Experiments on Artificial Synthetic Networks

In this section, two types of synthetic networks, namely Lancichinetti–Fortunato–Radicchi (LFR) [71] and Girvan–Newman (GN) [4], are used to test the performance of LPA-MNI. The results are compared with six algorithms introduced in Section 4.1, and NMI and AMI are treated as the evaluation metrics.

#### 4.3.1. Experiment on LFR Benchmark Networks

The LFR benchmark network that is proposed by Lancichinetti et al. [71] has similar heterogeneous characteristics to the real network, i.e. node degree and community size follow power law distribution. The LFR benchmark network is one of the most commonly used synthetic networks for measuring the performance of community detection algorithms. The LFR generator provides a set of parameters to produce different networks, including the number of nodes *N*, the average degree $\langle k\rangle$, the maximum degree Max(k), the mixing parameters μ, the minimum community size Min(c), and the maximum community size Max(c). In addition, the exponents for degree distribution and community size distribution are $\tau_{1}$ and $\tau_{2}$, respectively. The mixing parameter μ represents the link fraction that connects to other communities. The larger the mixing parameter, the less clear the community structure. The parameters of the LFR network used in the paper are set, as shown in Table 7. Eight groups of networks are generated, and each group consists of eight networks with mixing parameter μ ranging from 0.1 to 0.8 and other common parameters.

**Figure 8:**
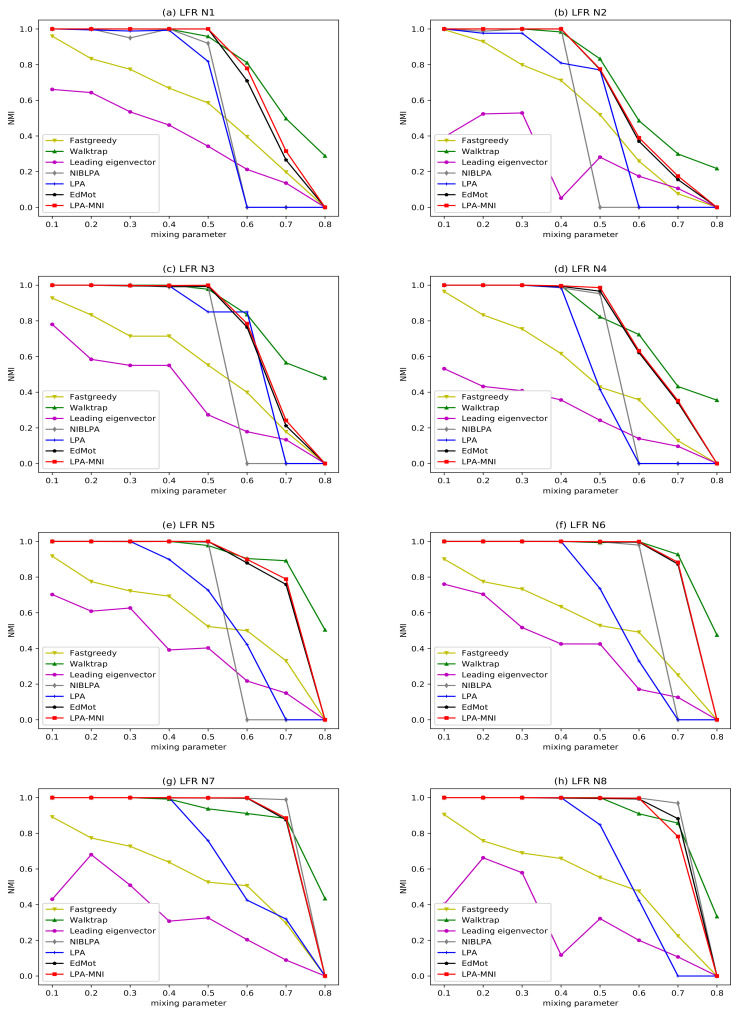
The NMI results of seven algorithms on LFR benchmark networks.

**Figure 9:**
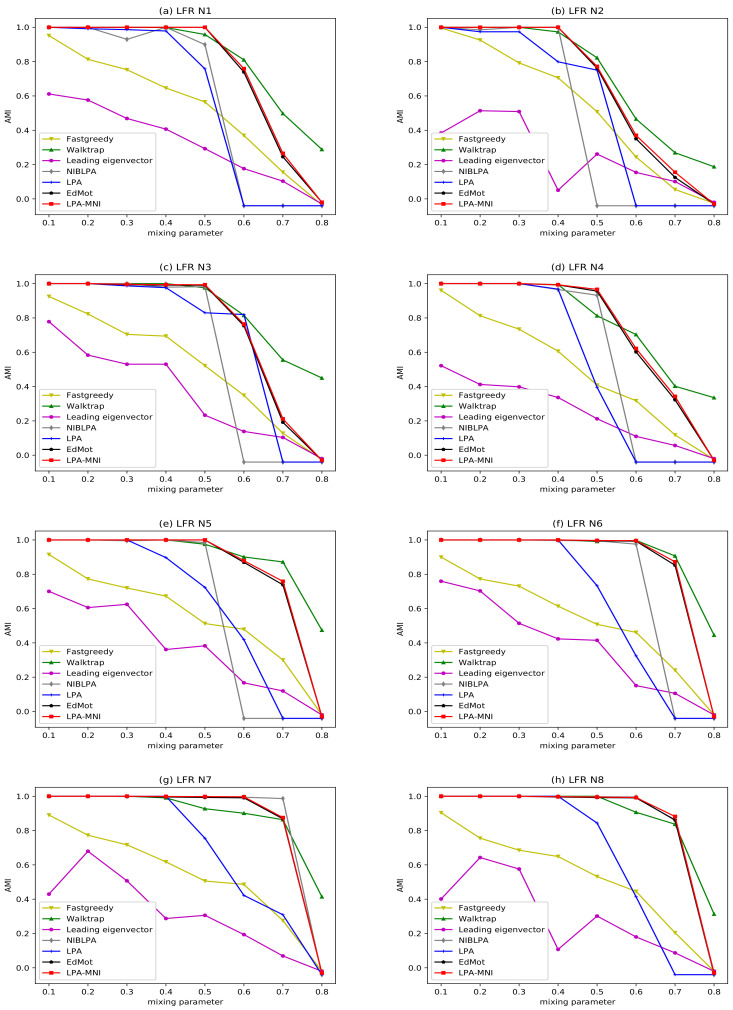
The AMI results of seven algorithms on LFR benchmark networks.

Figure 8 and Figure 9 illustrate the experimental results of LFR benchmark networks, and the result of LPA is the average value obtained by running 100 times. The results indicate that all algorithms have good performance, since the mixing parameter μ is small. With the increase of μ, the network becomes increasingly complex, which makes it more difficult to reveal the community. The LPA-MNI algorithm has stable and good performance on LFR N1-N4 under μ≤0.5, on LFR N5-N8 under μ≤0.7. However, the performance of Fastgreedy and Leading eigenvector decrease obviously when μ>0.1, and the performance of LPA drops significantly when μ≥0.5, which is same to NIBLPA. In all of the experiments, LPA-MNI algorithm generally outperforms other four compared algorithms LPA, Fastgreedy, Leading eigenvector, and NIBLPA in all datasets. Additionally, the EdMot algorithm obtains the comparable results with LPA-MNI algorithm. Although Walktrap has a slightly higher value of NMI when μ≥0.6, LPA-MNI can obtain better results on LFR N1-N4 under μ<0.7 and on LFR N5-N8 under μ<0.8.

#### 4.3.2. Experiment on GN Benchmark Networks

Girvan-Newman [4] proposed the GN benchmark network. Each network is composed of four communities and 32 nodes are in each community. Each node has the expected degree $p_{in}+p_{out}=16$, in which $p_{in}$ and $p_{out}$ donate the connection probability of internal and external nodes, respectively. That is to say, each node expected 16 links, $p_{in}$ links are connected to its own community, and $p_{out}$ links are randomly connected to other communities. With the increase of $p_{out}$, the community structure is becoming less obvious.

**Figure 10:**
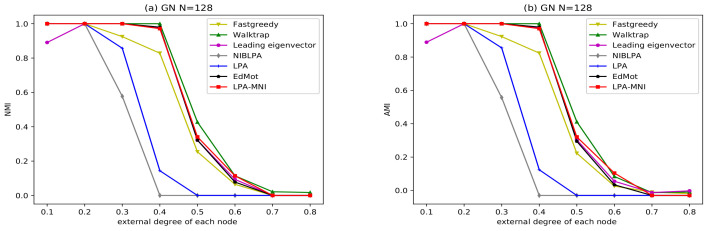
Comparisons of seven algorithms on GN benchmark networks.

With the increase of the external degree of each node, the ability of all algorithms to divide the community structure gradually decreases, as shown in Figure 10. When the parameter is greater than 0.6, all of the algorithms can not divide the community structure. When the parameter is less than or equal to 0.6, LPA-MNI algorithm has better performance than that of LPA, Fastgreedy, Leading eigenvector, and NIBLPA, and it is comparable to Walktrap and EdMot. In particular, it is a disappointment that the original LPA algorithm cannot distinguish any community when $p_{out}\geq 0.5$.

### 4.4. Comparison of Computational Complexity

Table 8 shows the computational complexities of LPA-MNI and other comparison methods. The experimental results show that LPA-MNI has significant improvement in stability when compared with the original LPA algorithm, but its time complexity does not increase. In addition, the time complexity of LPA-MNI is lower than Fastgreedy, Leading eigenvector, Walktrap, and EdMot algorithms. Although the time complexity of NIBLPA algorithm is linear, its performance is worse than that of LPA-MNI.

### 4.5. Critical Discussion

The networks that are adopted in the experiments are static networks that have been widely used for community detection. Therefore, the performance of proposed algorithm in dynamic network have not been tested. In the future, the problem of community detection in dynamic network will be further explored.

## 5. Conclusions

Traditional LPA is a classical algorithm with near linear time complexity for community detection. However, there is strong randomness in its partition results. Therefore, in this study, an improved algorithm LPA-MNI is proposed to detect the community structure in complex networks. The core idea of LPA-MNI is to combine the modularity and node importance to deal with the instability of LPA. 12 real-world networks and two types of synthetic networks are used to measure the performance of the LPA-MNI algorithm and the results are compared with six advanced algorithms. The experimental results show that the LPA-MNI algorithm can get closer results to the real partition in the networks with known community structure. Additionally, LPA-MNI can achieve higher modularity in the networks with unknown community structure. Furthermore, the comparisons indicate that LPA-MNI has better stability than LPA. Further research will focus on developing a more effective community detection algorithm for weighted, directed, and dynamic networks.

## Figures and Tables

**Table 1:** The topology features of real-world networks. |N| and |E| represent the number of nodes and edges, respectively. Max(k) is the maximum degree, 〈k〉 is the average degree, and *C* is the average clustering coefficient.

Networks	|N|	|E|	Max(k)	〈k〉	*C*
Karate [54]	34	78	17	4.588	0.256
Dolphins [62]	62	159	12	5.129	0.309
Football [4]	115	613	12	10.661	0.407
Riskmap	42	83	6	3.952	0.435
Lesmis [63]	77	254	36	6.597	0.499
Jazz [64]	198	2742	100	28.563	0.520
PolBlogs [65]	1222	16,714	351	27.355	0.226
Yeast [66]	2375	11,693	118	9.847	0.469
Ca_Hep [67]	9877	25,973	65	5.259	0.284
Astro-ph [67]	16,706	121,251	360	14.516	0.426
Cond_mat [68]	16,726	47,594	107	5.691	0.360
Cond_mat2005 [68]	40,421	175,692	278	8.693	0.650

http://en.wikipedia.org/wiki/Risk(game). Accessed on 1 March 2021.

**Table 2:** The actual community structure of karate network.

Community ID	Members
1	1,2,3,4,5,6,7,8,11,12,13,14,17,18,20,22
2	9,10,15,16,19,21,23,24,25,26,27,28,29,30,31,32,33,34

**Table 3:** The results of seven algorithms on karate network.

Algorithm	Fastgreedy	LPA	Leading Eigenvector	Walktrap	NIBLPA	EdMot	LPA-MNI
CN	3	2	4	5	3	3	**2**
Q	0.380	0.292 ± 0.292	0.393	0.353	0.352	**0.412**	0.372
NMI	0.692	0.585 ± 0.415	0.677	0.504	0.625	0.602	**1**
AMI	0.681	0.571 ± 0.403	0.661	0.473	0.618	0.581	**1**

**Table 4:** The results of seven algorithms on dolphins network.

Algorithm	Fastgreedy	LPA	Leading Eigenvector	Walktrap	NIBLPA	EdMot	LPA-MNI
CN	4	3	5	4	5	4	**4**
Q	0.495	0.492 ± 214	0.491	0.489	0.452	0.518	**0.527**
NMI	0.787	0.732 ± 0.210	0.679	0.692	0.721	0.830	**0.843**
AMI	0.773	0.722 ± 0.110	0.652	0.671	0.719	0.815	**0.833**

**Table 5:** The results of seven algorithms on football network.

Algorithm	Fastgreedy	LPA	Leading Eigenvector	Walktrap	NIBLPA	EdMot	LPA-MNI
CN	6	9	8	10	9	9	**11**
Q	0.549	0.576 ± 0.072	0.492	0.602	0.542	**0.604**	0.582
NMI	0.697	0.880 ± 0.114	0.698	0.887	0.707	**0.889**	**0.889**
AMI	0.650	0.866 ± 0.102	0.633	0.856	0.685	0.859	**0.870**

**Table 6:** The results of seven algorithms on real-world networks.

Network	Metrics	Fastgreedy	LPA	Leading Eigenvector	Walktrap	NIBLPA	EdMot	LPA-MNI
Riskmap	Q	0.625	0.534 ± 0.126	0.546	0.623	**0.634**	**0.634**	**0.634**
Lesmis	Q	0.501	0.348 ± 0.049	**0.532**	0.521	0.348	0.525	**0.527**
Jazz	Q	0.439	0.282 ± 0.105	0.394	0.438	0.293	**0.444**	0.415
PolBlogs	Q	0.426	0.418 ± 0.129	0.424	0.425	0.422	0.237	**0.427**
Yeast	Q	0.700	0.657 ± 0.020	0.628	0.677	0.660	**0.728**	0.678
Ca_Hep	Q	**0.716**	0.631 ± 0.045	0.583	0.663	0.628	0.669	**0.675**
Astro-ph	Q	0.633	0.551 ± 0.107	0.595	0.636	0.606	0.546	**0.687**
Cond_mat	Q	**0.778**	0.720 ± 0.016	0.588	0.741	0.698	0.745	**0.750**
Condmat_mat2005	Q	**0.631**	0.445 ± 0.173	0.359	0.599	0.558	0.626	**0.629**

**Table 7:** The parameters of the LFR benchmark networks.

Network	*N*	<k>	Max(k)	$\tau_{1}$	$\tau_{2}$	Min(c)	Max(c)	μ
LFR N1	1000	15	50	2	1	10	50	0.1–0.8
LFR N2	1000	15	50	2	1	20	100	0.1–0.8
LFR N3	2000	15	50	2	1	10	50	0.1–0.8
LFR N4	2000	15	50	2	1	20	100	0.1–0.8
LFR N5	5000	25	50	2	1	20	50	0.1–0.8
LFR N6	5000	25	50	2	1	20	100	0.1–0.8
LFR N7	10,000	25	50	2	1	20	50	0.1–0.8
LFR N8	10,000	25	50	2	1	20	100	0.1–0.8

**Table 8:** Comparisons of computational complexity.

Algorithm	Time Complexity
Fastgreedy	$O(nlog^{2}n)$
LPA	O(m)
Leading eigenvector	$O(n^{2})$
Walktrap	$O(n^{2}*m)$
NIBLPA	O(m)
EdMot	$O(m^{1.5}+n\log n)$
LPA-MNI	O(m+nlogn)

## Data Availability

The data presented in this study are openly available in http://www-personal.umich.edu/~mejn/netdata/ and www.arxiv.org/, accessed on 1 March 2021.
